# Self-injecting non-prescribed substances into vascular access devices: a case study of one health system’s ongoing journey from clinical concern to practice and policy response

**DOI:** 10.1186/s12954-022-00707-4

**Published:** 2022-11-24

**Authors:** Jocelyn Chase, Melissa Nicholson, Elizabeth Dogherty, Emma Garrod, Jocelyn Hill, Rupinder Brar, Victoria Weaver, William J. Connors

**Affiliations:** 1 Department of Medicine, University of British Columbia, Vancouver, Canada; 2 Division of Geriatric Medicine, St. Paul’s Hospital, Providence Health Care, Vancouver, BC Canada; 3 Providence Health Care, Vancouver, BC Canada; 4 British Columbia Centre On Substance Use, Vancouver, Canada; 5 Vancouver Coastal Health, Vancouver, BC Canada; 6 Inter-Department Division of Addiction Medicine, Providence Health Care, Vancouver, BC Canada; 7 Division of Infectious Diseases, St. Paul’s Hospital, Providence Health Care, Vancouver, BC Canada

**Keywords:** Substance use, Vascular access devices, Harm reduction, Medical ethics, Healthcare policy, Intravenous substance abuse

## Abstract

**Background:**

Overdose-associated deaths and morbidity related to substance use is a global public health emergency with devastating social and economic costs. Complications of substance use are most pronounced among people who inject drugs (PWID), particularly infections, resulting in increased risk of hospitalization. PWID often require intravenous access for medical treatments such as antibiotics; however, vascular access may be limited due to the impacts of long-term self-venipuncture. While vascular access devices including peripherally inserted central catheters (PICCs) allow reliable and sustained routes of administration for indicated therapies, the use of PICCs among PWID presents unique challenges. The incidence and risks associated with self-injecting non-prescribed substances into vascular access devices (SIVAD) is one such concern for which there is limited evidence and absence of formal practice guidance.

**Case presentation:**

We report the experience of a multidisciplinary team at a health organization in Vancouver, Canada, working to characterize the incidence, patient and healthcare provider perspectives, and overall impact of SIVAD. The case study of SIVAD begins with a patient’s perspective, including patient rationale for SIVAD, understanding of risks and the varying responses given by healthcare providers following disclosure of SIVAD. Using the limited literature available on the subject, we summarize the intersection of SIVAD and substance use and outline known and anticipated health risks. The case study is further contextualized by experience from a Vancouver in-hospital Overdose Prevention Site (OPS), where 37% of all individual visits involve SIVAD. The case study concludes by describing the systematic process by which local clinical guidance for SIVAD harm reduction was developed with stakeholder engagement, medical ethics consultation, expert consensus guideline development and implementation with staff education and planned research evaluation.

**Conclusion:**

SIVAD is encountered with enough frequency in an urban healthcare setting in Vancouver, Canada, to warrant an organizational approach. This case study aims to enhance appreciation of SIVAD as a common and complex clinical issue with anticipated health risks. The authors conclude that using a harm reduction lens for SIVAD policy and research can provide benefit to clinicians and patients by offering a clear and a consistent healthcare response to this common issue.

## Background

Vancouver, British Columbia, Canada, with a metropolitan population of 2.5 million in a province of 4.7 million people, is facing a public health emergency of drug overdose and associated death [1]. In 2021 alone, 535 Vancouver municipal and 2267 provincial illicit drug toxicity deaths were reported, representing the leading cause of unnatural death with an average 6 deaths per day [2]. It is well known that persons who inject drugs (PWID) are at increased risk of overdose and invasive bacterial infections such as infective endocarditis or osteomyelitis, with associated increases in hospitalization rates, morbidity and mortality compared to those who do not inject drugs [3–6].

Many of these bacterial infections require ongoing intravenous (IV) medications and vascular access devices (VADs). Peripheral intravenous catheters and peripherally inserted central catheters (PICCs) are clinically indicated in these circumstances. A PICC is often used when vascular access is limited or IV medications such as antibiotics are required for several weeks. Traditional peripheral VAD options may be limited in some PWID as a result of chronic venous disease induced by years of self-venipuncture, making PICCs an important (and sometimes necessary) consideration when protracted IV therapy is required [7].

Clinicians caring for patients who use substances in hospital and community settings, including Overdose Prevention Sites (OPS) and Supervised Consumption Sites (SCS) [8], may encounter patients self-injecting non-prescribed substances into their vascular access devices (SIVAD) rather than performing self-venipuncture. To date, the incidence of SIVAD and related complications has not been well studied, nor is it known if harm reduction measures reduce risks associated with SIVAD. No formal guidance has been issued regarding an optimal approach to this complex clinical issue from healthcare institutions, professional colleges, or harm reduction research and policy development bodies.

This paper describes a case study at Providence Health Care (PHC), an organization in Vancouver, British Columbia, where SIVAD is encountered with relative frequency. It begins with an exploration of SIVAD from the perspective of an individual patient, followed by data collected from a local Vancouver OPS and a review of the available literature in an attempt to characterize the clinical context, incidence and anticipated risks of SIVAD. The case study concludes by describing the systematic efforts of a multidisciplinary team in Vancouver to create and implement a local nursing clinical practice guideline and associated education and research plan around harm reduction approaches to SIVAD.

The authors also discuss current knowledge gaps and controversies around the medical, ethical and legal legitimacy of harm reduction for SIVAD and applicability of the Vancouver approach to other national and international contexts. This paper’s aim is to enhance the clinical understanding of SIVAD and through transparent shared experience, encourage other healthcare teams to develop and research SIVAD-related practices that are appropriate for their local context.

## Methods

This descriptive case study is comprised of multiple related research efforts sharing a common research objective: understanding SIVAD practice and impact at our health organization to inform development and implementation of related policy and practice. Rather than having a prespecified overarching research plan and methodology, this case study integrates mixed methods of multiple related projects identified and purposefully brought together through organization-wide efforts—risk management and ethics consultations—triggered in part by incident cases of SIVAD. The methodology of individual projects is further outlined in relevant sections.

### SIVAD patient perspective and clinical features

#### Patient perspective of SIVAD

Patient-centered care begins with exploring patient perspectives. As outlined in “Robin’s” case (see Table 1), hospitalization can be challenging for people with substance use disorders (SUD) as they may experience withdrawal symptoms and cravings following interruption of their usual substance use patterns [9]. Unmanaged pain, withdrawal and cravings may lead individuals to use non-prescribed substances while admitted to hospital [10]. Self-venipuncture options may be limited in some PWID because of venous thrombosis, sclerosis and occlusion of preferred injection sites in the upper and lower extremities [7]. A minority of PWID will inject into the internal jugular vein in the neck, the femoral vein in the groin, or smaller veins in the forehead or penis, which are often perceived as “higher risk” for complications, such as infection and excessive blood loss [11, 12].

**Table 1:** Patient experience and perspective of SIVAD

Robin is 38 years old and lives in Vancouver’s Downtown Eastside. She describes herself as a vibrant, active community member who has been injecting drugs for the last 14 years. She lost both of her parents in a tragic accident in her young adulthood, creating complex and strained family relationships. In her early 20’s, she started using stimulants and at age 25 began taking oxycodone for her osteoarthritis. At age 27, she began injecting drugs intravenously (IV). She receives injectable opioid agonist therapy (iOAT)^1^ at an outpatient clinic, but iOAT alone has not been sufficient to treat her pain and opioid tolerance, and she continues to use additional non-prescribed IV drugs
Five years ago, Robin was admitted to hospital with a skin infection. It was around this time that she had run out of veins in her arm that she could easily inject into. Thus, she started injecting substances into her abdomen, legs, upper arms and jugular vein. Frequent vein misses (“missed hits”) resulted in many areas of skin breakdown and abscesses. A peripherally inserted central catheter (PICC) was inserted for her to receive IV antibiotics
She remembers being in a lot of pain and watching the nurses clean and flush the line, attach syringes loaded with opioids, and administer the medication so easily. She thought “I could do that.” She started collecting pre-packaged saline flushes accessible around the hospital unit and was relieved not to have to inject into her neck, and to have a way to avoid severe “dope-sickness” (withdrawal)
During subsequent admissions to hospital, she disclosed her PICC use to nurses and physicians and asked for sterile supplies. Sometimes the IV team inserting the PICC informed her about the risks of using it, and sometimes she was given supplies and education on how to use/access the PICC using sterile technique. One healthcare provider told her “You’re going to kill yourself” by using the PICC, but she was not told how or why this may be true
On a recent hospital admission for another infection, her desired discharge plan was to receive IV antibiotics as an outpatient. Unfortunately, this was declined because she disclosed PICC use to healthcare providers. Instead, her IV antibiotics were switched to oral and her PICC removed. This came as a surprise to Robin, who felt as though she was being punished for her honesty. She does not recall anyone talking to her about this change in discharge plans. Because of this situation and based on variability in provider responses to her PICC line use, she no longer discloses her PICC use because she fears it will compromise relationships with healthcare providers, and ultimately, her healthcare
Robin says that she frequently sees patients at community OPSs injecting into vascular access devices using unsterile technique. Often, she will intervene and offer advice when she sees unsafe practices but worries about the lack of education among people who inject drugs
A review of Robin’s medical record reveals that she has received care from addiction medicine, infectious disease, internal medicine and wound care specialists during several recent admissions. Notes indicate that clinicians were aware of Robin’s PICC use, with some notes referring to “tampering” or “abuse” of the line. One provider noted that the patient was instructed by community workers on how to use her PICC to inject. There were no notes regarding patient-provider discussions around the risks of PICC use or teaching about sterile technique. She was given general education on overdose prevention. On several occasions, notes indicate that Robin left hospital with her PICC in place, despite the team’s plans to remove it prior to discharge

Patient perspective reflects a synthesis of medical records and multiple voluntary interviews with ‘Robin’ conducted by an addiction nurse educator during hospital admissions and community follow-ups between 2017 and 2020 as part of an ongoing patient experience exploratory study. Demographic details have been anonymized. Permission for publication was obtained and consent signed by the individual providing the above perspective

^1^*SIVAD* self-injection into vascular access device, ^2^*iOAT* injectable opioid agonist therapy. ^3^*PICC* peripherally inserted central catheter

As described by “Robin”, patients may perceive benefits arising from SIVAD such as avoiding self-venipuncture in less desirable or challenging sites, or maintaining venous access to avoid or alleviate drug withdrawal. Patients may or may not be aware of SIVAD related health risks and may receive a variety of responses from healthcare providers ranging from PICC removal and discharge to education on risks and provision of harm reduction supplies.

Just as “Robin” experienced, the majority of PWID with a history of hospital PICC insertion interviewed by Guta and colleagues for a recent qualitative study reported that they had been subjected to “threats of discharge”. Stigmatizing experiences were common. And similar to “Robin’s” observations about awareness of risk, many PWID may be unaware of the possible risks of SIVAD [13].

#### Potential infectious and non-infectious risks of SIVAD

As exemplified by “Robin’s” recurrent hospitalizations, PWID, independent of IV access or its use, are at increased risk of overdose and invasive bacterial infections [3, 4]. Additionally, VADs themselves have inherent risks. These risks are modified by device, patient, and care environment related factors. For context, among non-PWID with central venous catheters or PICCs, the estimated incidence of associated bloodstream infections (BSI) is between 0.5 to 5.8% and deep venous thrombosis 3%, over the time period that a catheter remains in place [14, 15]. VAD malposition is estimated to occur in up to 9.3% of patients, while device dysfunction occurs at rates up to 78 per 10,000 indwelling days [16]. VAD complications often require device interventions, such as occlusion management, repair, removal, and replacement [17, 18].

Mirroring “Robin’s” experiences in hospital, clinicians report concern about risks of using PICCs in PWID, especially in non-clinical environments, with some opting to remove PICCs and pursue second line oral antibiotic treatment for infections due to fears of liability and censure from colleagues [13]. While increased risk of VAD complication among PWID is often cited, empirical evidence demonstrating this is absent. Risk may be inferred and influenced by general conceptions about the risks of IV drug use (IDU) [19]. Where evidence exists, objective and perceived risk discordance becomes apparent.

Among a cohort of 159 PWID receiving extended courses of outpatient antibiotics via VAD, no significant differences in incidence of BSI, thrombosis, or complications warranting device removal were seen when compared with non-PWID in the same program [20]. A recent review of published studies evaluating the safety of outpatient parenteral (IV) antibiotics for PWID also found no difference in rates of IV access-related adverse events when compared with non-PWID [21]. There are limitations of the available data including lack of standardized assessment for and documentation of SIVAD incidence among study populations. Furthermore, selection bias for more medically and socially stable PWID to include in studies and non-standardized application of SIVAD prevention policies and harm reduction measures may limit generalizability of existing studies.

In summary, although the rates and types of complications associated with SIVAD have not been clearly defined, it could be anticipated that patients who engaged in SIVAD may experience serious harms, with some resembling the risks of traditional self-venipuncture (e.g. blood stream infection, overdose) and others unique to SIVAD (e.g. air embolism, thrombosis, loss of device functionality). Harms may be more likely if patients are unaware of best practices around VAD care including the need for sterile technique and proper routine flushing following injection of substances [22, 23]. The potential impact of SIVAD harm reduction measures, including education on risks, sterile technique, or provision of single-use, sterile supplies is unknown.

### Experience from Vancouver overdose prevention sites and supervised consumption sites

In the absence of formal practice guidelines, Overdose Prevention Sites (OPS) and Safe Consumption Sites (SCS) in Vancouver have developed informal harm reduction approaches to SIVAD. OPSs, like SCSs, are spaces where people can inject their own non-prescribed substances using single-use, sterile equipment. While SCSs are federally funded and have nursing staff on site, OPSs are typically staffed by peers (people with lived/living experience of substance use) and are provincially sanctioned as a temporary response to British Columbia’s drug overdose and death public health emergency [8, 24].

At Insite, an SCS in Vancouver’s Downtown Eastside neighborhood, staff report engaging in harm reduction activities around SIVAD since 2006 and have developed an internal nursing resource covering risk education (e.g. overdose, VAD dysfunction) and sterile self-injection procedures [25, 26]. Staff at Insite encourage clients to access veins via self-venipuncture or to inject into a muscle rather than use their VAD if present. Nurses also assess VAD function and for localized signs of infection. If a client elects to use their VAD despite education regarding risks, staff provide education on the safest possible SIVAD technique and sterile injection supplies. This informal nursing guideline is currently for internal use only and is not published or publicly accessible at this time.

In 2018, the St. Paul’s Hospital Overdose Prevention Site (SPH OPS) was opened [27, 28]. This OPS was located in a small trailer adjacent to the hospital, staffed by peer workers and was accessible to both hospital inpatients and community members. Between its opening in May 2018 and February 2021, there were 34,229 visits to this OPS of which 4931 (14%) were hospital inpatients and 1318 (27%) of patient visits involved SIVAD. A visit is defined by one individual encounter with a client, with some clients visiting multiple times per day to inject substances. Clients used identification “handles” instead of names so it is not possible to attribute the number of SIVAD visits to specific individuals (i.e. if an individual visits frequently to use their VAD, each visit is counted towards the total visits involving SIVAD). SIVAD was recorded whenever a peer noted a patient accessing their VAD to inject drugs.

When the OPS initially opened, peers reported witnessing a variety of approaches to SIVAD including, but not limited to, using water from a water bottle to flush VADs, not cleaning the PICC hub prior to injection and inserting needles into the IV tubing itself as opposed to the access port [29]. At the same time, clinical staff inside the hospital reported to Clinical Nurse Educators that hospitalized patients were engaging in SIVAD on inpatient units, often in unsupervised settings.

Due to these observed safety concerns, quality improvement measures were instituted at the SPH hospital-adjacent OPS and other Vancouver OPSs. Since 2019, peers receive standardized education on how to provide harm reduction interventions for SIVAD through a “street degree” program and OPSs stock supplies specific to SIVAD (e.g. pre-filled syringes with sterile flush solution, needleless syringes) [30]. Hospital and community patients known to be engaging in SIVAD are directed to OPSs for supervision and supplies.

### Systematic approach to create standardized clinical guidance for SIVAD and address knowledge gaps

In response to observed SIVAD in Vancouver acute and community care settings, as well as growing SCS/OPS experience offering SIVAD harm reduction education and supplies, a multidisciplinary healthcare team at Providence Health Care (PHC) took steps in 2018 to examine SIVAD more formally. PHC is a health organization in Vancouver that administers care to approximately 600,000 patients annually via two acute care hospitals with approximately 500 acute beds in total. The goal of this work was to develop consistency in the response to SIVAD through a practice guideline based on available evidence and expert consensus, as well as to identify SIVAD as an issue worthy of formal clinical response, education and research.

#### Incident cases and risk management engagement

Instigated by a series of distressing SIVAD incidents noted by healthcare providers (e.g. patients performing SIVAD in unsupervised and unsafe environments such as medical ward shower stalls), the organization’s Risk Management and Patient Safety program commissioned a SIVAD risk analysis review. A group of Infectious Diseases, Urban Health and Addiction Medicine specialist physicians, nurses, clinical ethicists and the organization’s lawyer, was assembled to discuss potential patient, healthcare provider, institutional and wider community implications of SIVAD and potential harm reduction policy interventions [31]. A failure modes effect analysis (FMEA) was conducted to systematically identify and evaluate the anticipated risks of SIVAD and the impacts of potential adverse outcomes following a policy change (‘failure modes’), in this case harm reduction interventions for SIVAD [32].

The FMEA ranked priorities to be anticipated and addressed when considering policy development and monitoring. The most concerning risk anticipated to arise following harm reduction implementation was increased SIVAD rates amongst the larger community of PWID (not just in those specifically educated) should it be misperceived that “clinicians now condone the use of VADs for self-injection”. VAD access dysfunction was the next most concerning risk. Somewhat surprisingly, participants articulated less concerns about risk of infectious complications and overdose. This may reflect acknowledgement of baseline risks associated with IDU independent of SIVAD and an understanding that a SIVAD policy or guideline would intentionally aim to mitigate such harms.

#### Formal organizational SIVAD ethics consultation

Building from the FMEA, a more in-depth investigation was required to explore the implications of possible practice changes around SIVAD. In November 2019, the organization’s Ethics Services program was consulted by the Urban Health program to review the following questions:

1. Will the organization support the development of a policy/guideline that permits clinicians’ discretion to deliver SIVAD harm reduction education and supplies to patients with substance use disorders upon request, while also allowing for conscientious objection should clinicians wish to opt out of the practice?
2. What ethical, medical and organizational considerations ought to be considered with this approach?

Following approval from the Senior Leadership Team in February 2020, Ethics Services conducted an organizational ethics consultation that took 6 months to complete. Organizational ethics is the discipline concerned with the principles and standards by which an organization operates. It focuses on finding the “right” way to respond to complex challenges and opportunities within the communities that the organization serves [33]. Because providing SIVAD harm reduction was recognized to be a response that could significantly impact the lives of patients, clinicians and the organization’s reputation, formal ethical reflection was requested given limited medical evidence on the subject and a general absence of practice standards.

A total of 50 stakeholders were interviewed, including patients with lived/living experience of drug use/SIVAD, program leaders, physicians and nurses working in acute care or community in Vancouver, as well as clinicians at other Canadian sites. An interview question guide was developed by Ethics Services in consultation with a Clinical Nurse Educator in Substance Use. Interviews were one hour long, semi-structured, non-remunerated and occurred in person or over videoconferencing, either individually, or in small groups of up to 4 participants per participants preference. Several individuals provided information only over email. We identified stakeholders by contacting program leaders in Infectious Disease, Internal Medicine, Urban Health, Addiction Medicine, Psychiatry, IV Therapy, Risk Management, and Nursing Professional Practice. Additional stakeholders were identified during the interviews themselves (i.e. “who else should we speak to about this question?”). Stakeholder responses were organized into themes (see Table 2). No other local or national center was found to have formal organizational guidelines or policies on SIVAD harm reduction.

**Table 2:** Stakeholder Perspectives from the Organizational Ethics Consult. *Source* Providence Health Care Ethics Services Organizational ethics consult: Harm reduction, an approach for patients who self-inject non-prescribed substances into their vascular access devices, December 2020

*Patient Stakeholders*
• Identify personal experience with self-injection of non-prescribed substances into vascular access devices (SIVAD), but an incomplete awareness of risks, and interest in more education
• Endorse benefits of SIVAD that may not be valued by healthcare providers (e.g. avoiding “high risk” venipuncture, better management of drug withdrawal, and stabilizing substance use disorder enabling completion of medical treatment)
*Nursing Stakeholders*
• Indicate SIVAD harm reduction appears “common sense” and patient-centered
• Some already engage in SIVAD harm reduction, while others are uncertain and desire more education and organizational guidance and support
• No nurses interviewed expressed objection to SIVAD harm reduction but noted that some nurses will object based on moral or philosophical grounds, or because of lack of experience or evidence
*Physician Stakeholders*
• Desire organizational support should a legal challenge arise following an adverse event
• Infectious Diseases physicians endorse experience with infectious complications of SIVAD
• Some believe that SIVAD should prompt vascular access device removal and switch to oral antibiotics
• Some believe that shared informed decision making would be helpful in patients who SIVAD
*Risk Management and Professional Practice*
• Documenting a discussion on anticipated risks of SIVAD would satisfy the need for informed consent, and could mitigate legal risk to individuals and the organization
• Legal liability coverage for nurses is provided by the organization
• Harm reduction interventions are within nursing scope to provide

Following a detailed ethical analysis, the Ethics Services team determined that it would be ethically permissible to create an organizational harm reduction guideline around SIVAD. In light of the risks of harm—that is, the degree of severity and relative certainty of harms related to overdose and drug-poisoning deaths when SIVAD occurs covertly and without supervision, sterile supplies or education—the ethics team identified proportionate rationale and clear ethical justification to move forward with supervised SIVAD harm reduction, particularly within a monitored setting such as an OPS, with organizational oversight including research and dedicated staff education. With an overarching goal to save lives and engage patients in care, harm reduction strategies were believed to be appropriate as part of the comprehensive program of services PHC provides for people who use substances.

Importantly, given the general lack of directive evidence in this area, providers who do not feel comfortable with SIVAD harm reduction could opt out. See Table 3 for specific consult recommendations, which were reviewed and approved by the institution’s Risk Management department and Senior Leadership Team in February 2021.

**Table 3:** Recommendations from the organizational ethics consult. *Source* Providence Health Care Ethics Services Organizational ethics consult: Harm reduction, an approach for patients who self-inject non-prescribed substances into their vascular access devices, December 2020

1. At a minimum, develop a patient education intervention around the risks of self-injection of non-prescribed substances into vascular access devices (SIVAD)
2. Consider an interim SIVAD harm reduction guideline for use in the organization, with expert stakeholder input, and an opt-out option for providers who disagree
3. Continue to develop and promote wraparound care for patients with substance use disorders (Addiction Medicine consultation, social work, and overdose prevention site (OPS) models of care)
4. Study the incidence and outcomes of SIVAD harm reduction within the organization
5. Use a standardized SIVAD chart document to demonstrate patient informed consent
6. Position SIVAD harm reduction in an OPS environment initially to ensure consistency, quality, and research opportunities
7. Develop an education program for clinicians around harm reduction generally, and practices and patient counselling techniques specific to SIVAD
8. Consider additional legal/risk evaluation regarding SIVAD harm reduction
9. Involve partner organizations in the development of guidelines, given the possible impacts to the community

#### SIVAD nursing harm reduction guideline development

The Senior Leadership Team’s endorsement of the Ethics Consultation recommendations was instrumental to support the development of a harm reduction guideline for SIVAD. Although intended as guidance for all healthcare providers, the document was created primarily as a nursing guideline, as it was acknowledged that nurses are the frontline providers of harm reduction education and supplies. Given that SIVAD and the impact of potential harm reduction is an emerging area of study, the guideline was positioned as an internal interim document, subject to future review and amendments as new information becomes available.

To create the guideline, a nurse educator with experience in substance use convened a working group of stakeholders that included nurses, patient care managers, physicians, educators and social workers representing disciplines of Addiction Medicine, Infectious Diseases, and Urban Health. The group utilized the limited research evidence available (including sources referenced in the FMEA and Ethics Consult), existing organizational guidelines related to general VAD care and maintenance and information regarding strategies employed by other sites (including community run OPSs/SCSs as described above and a community OPS in Ottawa, Ontario) [34, 35].

A common concern raised by frontline nurses was potential legal liability if a nurse inadvertently flushed an IV line containing substances previously injected by a patient, resulting in patient overdose. Risk Management indicated that nurses are not liable for autonomous decisions made by capable patients, including use of substances and/or if patients experience an adverse event attributed to SIVAD. The importance of documenting an informed discussion with patients regarding risks of SIVAD was emphasized. As the organization takes a more formal harm reduction approach to SIVAD, liability coverage is in place provided nurses are practicing at the expected standard and this has been indicated within the guideline itself.

The group also consulted PHC’s Nursing Professional Practice consultants and the British Columbia College of Nurses and Midwives about nursing scope of practice pertaining to harm reduction and SIVAD specifically. These bodies endorsed that harm reduction practices, including providing education and sterile supplies, are within the scope of nursing practice [36]. Importantly, they indicated that nurses can act independently of physician oversight when assessing patients and implementing harm reduction measures within scope, however, conferral with the multidisciplinary care team is encouraged to problem solve around addiction management and medical care (see Table 4).

**Table 4:** Clinical practices around SIVAD that can be provided to patients by nurses as outlined in the clinical nursing practice guideline

1. Engage in standardized and comprehensive discussion on the risks of self-injection of non-prescribed substances into vascular access devices (SIVAD)
2. Primarily discourage SIVAD and encourage use of alternate injection techniques (e.g. venipuncture, muscular injection)
3. Complete a standardized documentation template (located in the hospital electronic medical record) that an informed discussion took place
4. If the patient decides to engage in SIVAD despite risks and alternate route suggestions, offer education on safer sterile injection techniques and provide sterile supplies, including saline flushes and alcohol swabs
5. Facilitate nursing communication about SIVAD activities to other providers, including Addiction Medicine and Infectious Disease clinicians, so that ongoing medical indication for the VAD can be assessed, and substance use disorder, withdrawal and cravings can be assessed and treatment optimized

The guideline also emphasizes that nurses have the ability to exercise discretion as to whether they will provide SIVAD education and sterile supplies to patients. If nurses choose to opt-out of this practice (e.g. concerns over the absence of evidence for SIVAD, moral objections, or clinical concerns related to patient factors), they are advised to transfer patient care to another clinician who can assess the patient. Nurses can also consult addiction medicine or more experienced colleagues and/or direct patients to the in-hospital OPS where this care is routinely offered.

The revised version of the guideline was approved and posted within the organization. The guideline is clear that providing supplies and education does not equate to condoning SIVAD or substance use in hospital and patients are directed to the in-hospital OPS. The guideline also formalizes clinical practices around SIVAD that were already being *informally* provided to patients by nurses on a case-by-case basis in hospital as well as at community SCS/OPSs over the last several years.

#### SIVAD nursing harm reduction guideline implementation at the SPH OPS

At the end of 2020, the hospital-adjacent OPS moved to a new location which created a gap in service for hospital inpatients. In response, SPH opened an in-hospital OPS in February 2021 [37]. It is staffed by nurses who receive specialized training on providing harm reduction education and sterile supplies. The opening of the in-hospital OPS roughly coincided with the release of the interim SIVAD nursing harm reduction guideline, as outlined above. As such, SIVAD harm reduction training is provided to nurses based on the guideline and supplemented by education from an IV Therapy nurse specialist (e.g. assisting clients with alternate vein identification and vein care) [34].

Between its opening on February 1st to October 23, 2021, the site has had 1,655 visits, 611 (37%) of which involved SIVAD, which is recorded whenever a nurse notes a patient accessing their VAD to inject substances. Staff anecdotally report that SIVAD harm reduction education is the most common education requested by patients, many of whom already express existing knowledge about harm reduction practices for self-venipuncture [38].

#### Staff education on harm reduction and SIVAD

Nurse educators specialized in the care of patients with substance use disorders presented the new guideline at in-service sessions across acute care settings. The sessions equip nurses with skills to engage in harm reduction counselling with patients (e.g. language to use, trauma-informed care, information on medical risks) and to ensure that nurses understand the purpose and limitations of harm reduction. The guideline was also presented to nursing leadership at frontline nursing meetings across the organization so that nurses could bring this information back to their colleagues.

To enhance the reach of SIVAD-specific guidance, presentations at departmental rounds have been an important avenue for ongoing discussion and education. The British Columbia Centre on Substance Use hosts a monthly “What’s New in Addiction Medicine?” lecture series that is attended by a diverse range of healthcare professionals provincially. A rounds presentation reviewing SIVAD literature and approaches to VADs in PWID was delivered in May of 2021. This presentation had 65 live attendees and 102 registrants with access to online material [39].

#### SIVAD research in progress at providence health care, Vancouver

In line with the organizational ethics consult recommendations, research has been prioritized. Ethics approval has been obtained for a mixed-methods study of the attitudes, beliefs and practices of patients who engage in SIVAD and is currently in recruitment and data collection phases. Investigators are exploring patients’ understanding of SIVAD-associated risks (e.g. infection, air embolism), personal beliefs (e.g. why patients are using VADs to inject) and influencing factors (e.g. how the practice is being learned). Investigators will also conduct chart reviews to evaluate patient outcomes, including incidence of blood stream infections, thrombosis, occlusion, VAD malposition or accidental removal. Research to determine prevalence of SIVAD in an existing community study cohort of PWID is also underway.

## Discussion

There is evidence that a patient-centered, harm reduction approach to care helps PWID engage in healthcare and reduces overall harms of IDU [27, 40–42]. Harm reduction does not condone, endorse, or condemn substance use. Rather, it recognizes substance use as a reality for some individuals and focuses on reducing its harmful consequences [43]. PWID often inject in non-sterile, unsupervised settings due to stigma and criminalization, but when supported in safe environments, rates of IDU-related infections and fatal overdose decrease [44–46]. Harm reduction strategies ideally exist within a continuum of care, giving patients the option of accessing various social and medical supports that may improve their overall health [47, 48].

For patients who are informed of the anticipated risks associated with SIVAD but continue to endorse a plan to use their VAD, harm reduction interventions, including counselling about sterile technique and flushing and provision of sterile supplies is philosophically, ethically and medically analogous to sterile needle exchange/availability programs. If patients fear judgement from healthcare providers about SIVAD, they may engage in SIVAD covertly to avoid consequences such as VAD removal and premature discharge from hospital. Loss of patient engagement and concerns about overdose and adverse events up to and including death must be acknowledged. Harm reduction interventions, including safer injection techniques for non-prescribed substances helps support safer drug use patterns, access to primary care and reduced overdose frequency [49]. It is therefore plausible that supporting SIVAD harm reduction will lead to improved patient outcomes. Acknowledging that some providers may have moral distress in relation to SIVAD associated care—as highlighted in our case study—ongoing and targeted education about the purpose of SIVAD harm reduction and the existence of care pathways linking both patients and providers to skilled providers of such services must be central to any implementation plan.

There may be concern among healthcare providers that inserting PICCs or providing education on SIVAD to PWID may lead to increased IV drug use or SIVAD. Data is currently being collected and analyzed with respect to the incidence of SIVAD at the in-hospital SPH OPS following the introduction of the nursing guideline, to ascertain trends in SIVAD over time. It should be noted that the percentage of visits involving SIVAD rose from 27% in the hospital-adjacent OPS (May 2018–January 2021) to 37% at the in-hospital SPH OPS (February–October 2021). The significance of this observation requires more detailed analysis accounting for confounders and clinical outcomes. Populations accessing community and hospital-adjacent OPSs and those visiting in-hospital OPS are dissimilar and difficult to compare.

In order to mitigate the risk of increased SIVAD among populations of PWID following the introduction of harm reduction measures, it is important that any policies or guidelines developed are clear that the main goal of SIVAD harm reduction counselling is to provide education regarding potential risks and to discourage the patient from using their VAD to inject substances. Guidelines should be clear that providing supplies and education does not mean substance use or SIVAD is condoned.

Ongoing data collection to establish baseline incidence of SIVAD among PWID in inpatient and outpatient settings and documenting the type and rate of complications are needed to understand incidence and impact of SIVAD. Furthermore, qualitative assessment of patient experiences and perspectives regarding SIVAD are needed to contextualize this data and understand how it intersects with overall health and substance use. While a study in Vancouver is underway to begin to delineate these aspects of SIVAD, larger more inclusive multicenter collaborative research evaluating SIVAD is needed to appropriately account for heterogeneity of substance use behaviors, health system resources, policies and practices. Studies relating to PICC use and complications in PWID should include consideration of SIVAD, including creating standard means of identifying and documenting SIVAD in research protocols.

Assessment of the impacts of possible SIVAD harm reduction interventions (e.g. education on risks and sterile technique, provision of sterile supplies) are also needed. Such harm reduction-oriented studies should include evaluation of the rates and types of complications, while also exploring impact on patient quality of life, engagement in healthcare and substance use trajectories. Concurrent guidance and research, rather than a typical sequential approach of research followed by policy development should be considered, reflecting urgency arising from the ongoing overdose crisis in BC and elsewhere [50, 51].

Vancouver represents a unique intersection of patient population and practice landscape, where addiction medicine services and harm reduction interventions are widely accepted and available, including SCS/OPSs. The generalizability of the work described herein may be limited or of low priority in settings without such resources and research opportunities may be scarce in settings where SIVAD incidence is believed to be low.

## Conclusion

SIVAD poses complex clinical and ethical challenges that require efforts to close knowledge gaps towards the creation of guidelines and policies that support patients, clinicians and organizations in reducing harms and improving health outcomes for PWID. Although healthcare providers and organizations may prefer that patients avoid SIVAD, clinicians must be willing to engage with patients within the reality that currently exists. Much work remains ahead to establish a foundation of evidence and organizations should actively seek opportunities within their own institutions and multidisciplinary teams to study and formalize guidelines and policies around this important area of practice, reviewing and amending them as new evidence becomes available. As organizations gain experience, the publication of guidelines and collaborative efforts between institutions will be essential.

## Data Availability

The St Paul’s Hospital Overdose Prevention Side datasets presented are not publicly available as they include internal organizational documentation and program statistics but are available from the corresponding author upon reasonable request.

## Abbreviations

BSI: Bloodstream infections
FMEA: Failure modes effect analysis
IV: Intravenous
IDU: IV drug use
OPS: Overdose prevention site
PHC: Providence health care
PICC: Peripherally inserted central catheter
PWID: Persons who inject drugs
SCS: Supervised consumption site
SPH: St. Paul’s hospital
SPH OPS: St. Paul’s hospital overdose prevention site
SUD: Substance use disorder
SIVAD: Self-injection of non-prescribed substances into vascular access devices
VAD: Vascular access device
